# Potential Antioxidant Activity of Apigenin in the Obviating Stress-Mediated Depressive Symptoms of Experimental Mice

**DOI:** 10.3390/molecules27249055

**Published:** 2022-12-19

**Authors:** Adel Alghamdi, Mansour Almuqbil, Mohammad A. Alrofaidi, Abdulhadi S. Burzangi, Ali A. Alshamrani, Abdullah R. Alzahrani, Mehnaz Kamal, Mohd. Imran, Sultan Alshehri, Basheerahmed Abdulaziz Mannasaheb, Nasser Fawzan Alomar, Syed Mohammed Basheeruddin Asdaq

**Affiliations:** 1 Department of Pharmaceutical Chemistry, Faculty of Clinical Pharmacy, Al Baha University, P.O. Box 1988, Al Baha 65528, Saudi Arabia; 2 Department of Clinical Pharmacy, College of Pharmacy, King Saud University, Riyadh 11451, Saudi Arabia; 3 Department of Pharmacology, Faculty of Medicine, King Abdulaziz University, Jeddah 21589, Saudi Arabia; 4 Department of Pharmacology & Toxicology, College of Pharmacy, King Saud University, Riyadh 11451, Saudi Arabia; 5 Department of Pharmacology and Toxicology, Faculty of Medicine, Umm Al-Qura University, Al-Abidiyah, P.O. Box 13578, Makkah 21955, Saudi Arabia; 6 Department of Pharmaceutical Chemistry, College of Pharmacy, Prince Sattam Bin Abdulaziz University, Al-Kharj 11942, Saudi Arabia; 7 Department of Pharmaceutical Chemistry, Faculty of Pharmacy, Northern Border University, Rafha 91911, Saudi Arabia; 8 Department of Pharmaceutical Sciences, College of Pharmacy, AlMaarefa University, Ad Diriyah 13713, Saudi Arabia; 9 Department of Pharmacy Practice, College of Pharmacy, AlMaarefa University, Dariyah, Riyadh 13713, Saudi Arabia; 10 Equame Scientific and Research Center, Riyadh 13713, Saudi Arabia

**Keywords:** apigenin, antidepressant activity, antioxidant potential, chronic mild stress, sucrose preference test, tail suspension test

## Abstract

This study aimed to examine the antidepressant properties of apigenin in an experimental mouse model of chronic mild stress (CMS). Three weeks following CMS, albino mice of either sex were tested for their antidepressant effects using the tail suspension test (TST) and the sucrose preference test. The percentage preference for sucrose solution and the amount of time spent immobile in the TST were calculated. The brain malondialdehyde (MDA) levels, catalase activity, and reduced glutathione levels were checked to determine the antioxidant potential of treatments. When compared to the control, animals treated with apigenin during the CMS periods showed significantly shorter TST immobility times. Apigenin administration raised the percentage preference for sucrose solution in a dose-dependent manner, which put it on par with the widely used antidepressant imipramine. Animals treated with apigenin displayed a significantly (*p* ˂ 0.05) greater spontaneous locomotor count (281) when compared to the vehicle-treated group (245). Apigenin was also highly effective in significantly (*p* ˂ 0.01) lowering plasma corticosterone levels (17 vs. 28 µg/mL) and nitrite (19 vs. 33 µg/mL) produced by CMS in comparison to the control group. During CMS, a high dose (50 mg/kg) of apigenin was given, which greatly increased the reduced glutathione level while significantly decreasing the brain’s MDA and catalase activity when compared to the control group. As a result, we infer that high doses of apigenin may have potential antidepressant effects in animal models via various mechanisms.

## 1. Introduction

A mental condition known as depression is characterized by low mood, loss of interest in routine tasks, anhedonia, feelings of worthlessness, trouble sleeping, and suicidal thoughts [1]. The primary reasons include alterations in monoamine neurotransmitters [2], as well as increased oxidative and nitrosative damage [3]. Antioxidant levels were reported to decrease, and oxidative and nitrosative stress was found to be high in patients with depressive symptoms [4].

Depression impairs glucocorticoid response and elicits the hypersecretion of corticotropin-releasing hormone [5]. The hypothalamic-pituitary-adrenal axis is hyperactive in about 50% of depressed patients. When animals experience prolonged stress, the hyperactivity of the hypothalamic-pituitary-adrenal axis is altered [6]. Stress substantially impacts how depression manifests in people [7]. When experimental animals experience chronic mild stress (CMS), depressive symptoms develop. The development of chronic, stress-induced depression may be influenced by elevated brain oxidative stress brought on by stress, which is a significant contributor to neurological damage and nerve destruction [8,9].

In treating depression, antidepressants produce their therapeutic effect by their action on central monoaminergic systems, particularly through serotonergic and nor-adrenergic synaptic neurotransmission. Selective serotonin reuptake and specific serotonin-noradrenaline reuptake inhibitors are the most frequently prescribed medications for depression [10]. Even though they are effective in treating most depressive episodes, a sizable portion of depressed people does not exhibit indications of improvement until two to three weeks after beginning treatment. A third of these patients only partially or never react to medicine [11]. Additionally, these drugs may result in drowsiness, anticholinergic effects, seizures, impotence, postural hypotension, anxiety, vertigo, respiratory problems, weight gain, cheese reactions, cardiac dysrhythmias, sleeplessness, agitation, and exhaustion [10]. As a result, one option is to investigate the antidepressant efficacy of plant-based natural substances and their bioactive components.

A well-researched phenolic substance called apigenin (5,7,4′-trihydroxyflavone) is one of the most pervasive flavonoids in plants. Apigenin is primarily glycosylated in daily foods and nutritious beverages [12]. Apigenin shows potential as a nutraceutical and has several intriguing pharmacological activities. Its antioxidant properties are well known, and its potential therapeutic action has been established to treat conditions like autoimmune illness, cancer, inflammation, and neurological disease [13]. Due to the wide range of pharmacological effects and the importance of apigenin to human health, a thorough understanding of its mechanism of action is essential for its use in future nutraceuticals. 

Apigenin is well known for its relaxing and anti-anxiety properties [14,15]. Recent research validated apigenin’s ability to relax muscles in animal models [16]. According to research published by Nakazawa et al. [17], the dopaminergic system is involved in the possible antidepressant effect of apigenin. At the same time, another study demonstrated the role of α-adrenergic, dopaminergic, and 5-HT3 serotonergic receptors in mediating the antidepressant action of apigenin [18]. Monoamine oxidase (MAO) activity was likewise reduced by apigenin [19]. The inhibition of MAO increases levels of monoamines such as serotonin in the brain, which is linked to the disappearance of depressive symptoms [20]. The functioning of gamma-aminobutyric acid (GABA) and N-methyl-D-aspartate (NMDA) receptors has been shown in several cases to be inhibited by apigenin, and antagonizing these receptors may relieve depression [17,21]. 

The existing literature indicates that many neurotransmitters and processes are involved in the pharmacological activity of apigenin. There is, however, no research describing the pharmacological potential of apigenin under stress. It is widely acknowledged that chronic stress plays a role in the emergence of depression. We thought it would be worthwhile to investigate the connection between apigenin’s antioxidant capacity and its antidepressant properties, which are mediated by chronic stress, since depression is thought to be caused by a decline in antioxidant capacity, an increase in oxidative stress, and the presence of chronic stress. Therefore, a unique step is needed to identify perhaps another mechanism in the antidepressant efficacy of apigenin by correlating stress-induced depression with antioxidant potential. Consequently, this research was done to examine the possible role of apigenin in the treatment of mild chronic stress-induced depression, as well as the effects of apigenin on antioxidants in the brain.

## 2. Results

### 2.1. Immobility Time

**Figure 1:**
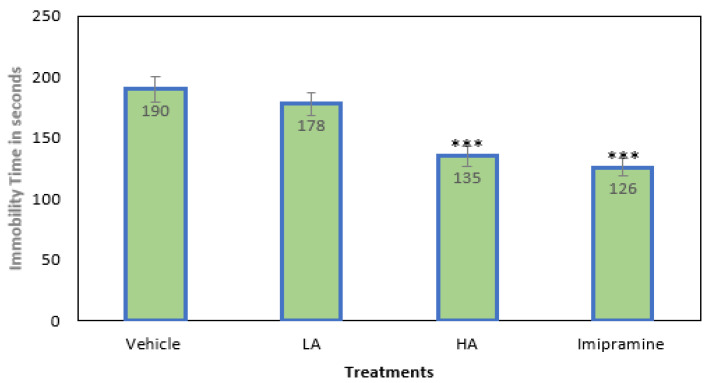
The effect of apigenin and imipramine on immobility period. Data are expressed in mean ± SEM (n = 8). Data were evaluated using a one-way analysis of variance (ANOVA) and Tukey’s test. *** *p* < 0.001 when compared to the vehicle control group. LA: low dose of Apigenin (25 mg/kg); HA: high dose of Apigenin (50 mg/kg); Imipramine: standard antidepressant (15 mg/kg); Vehicle; 0.2% carboxy methyl cellulose (CMC).

The animals in all four groups were subjected to mild chronic stress to induce depression. The duration of immobility was slightly reduced, but not significantly, by low doses of apigenin (25 mg/kg for LA). A considerable depletion in immobility time was seen following the administration of a high (*p* ˂ 0.001) dose of apigenin (50 mg/kg for HD) (Figure 1). The immobility period was also significantly (*p* ˂ 0.001) reduced by the standard tricyclic antidepressant imipramine (15 mg/kg), which was on par with the high dose of apigenin.

### 2.2. Sucrose Preference Model

**Figure 2:**
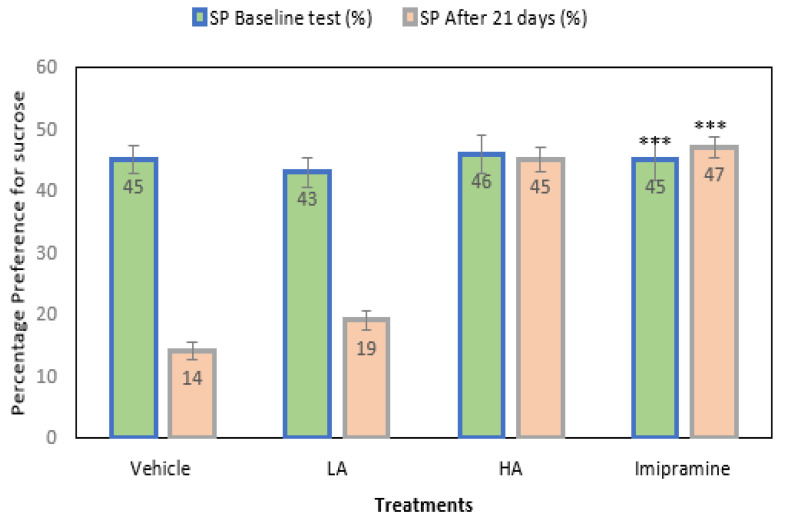
The effect of apigenin and imipramine on percentage sucrose preference. Data are expressed in mean ± SEM (n = 8). Data were evaluated using a one-way analysis of variance (ANOVA) and Tukey’s test. *** *p* < 0.001 when compared to the vehicle control group. LA: low dose of Apigenin (25 mg/kg); HA: high dose of Apigenin (50 mg/kg); Imipramine: standard antidepressant (15 mg/kg); Vehicle; 0.2% carboxy methyl cellulose (CMC).

The mice’s preference for sucrose was noted before and after the CMS’s end. The initial result showed no significant differences across the groups (Figure 2). Further, after 21 days of CMS, the vehicle-treated groups showed a significant (*p* ˂ 0.001) drop in their preference for sucrose relative to their baseline values. The animals’ preferences for sucrose decreased significantly (*p* ˂ 0.001) from their first response, despite apigenin being administered at a low dose. The administration of a high dose of apigenin and imipramine significantly (*p* ˂ 0.001) increased the preference of mice for sucrose compared to the group that received a vehicle treatment. In addition, there was no discernible change in these groups’ preferences for sucrose from their initial readings before the commencement of CMS.

### 2.3. Locomotor Activity 

**Figure 3:**
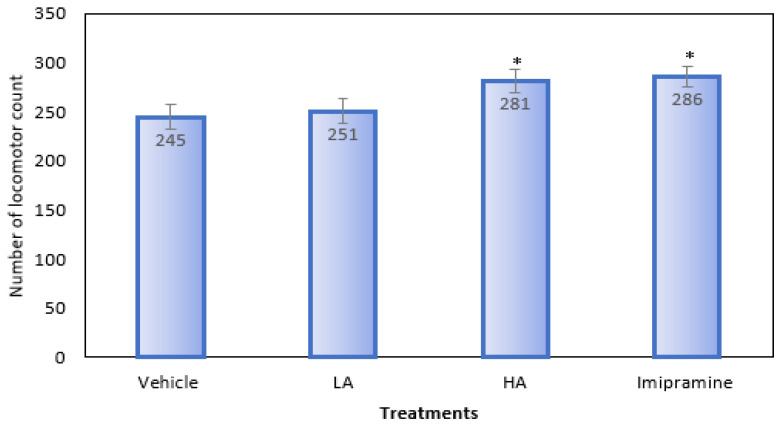
The effect of apigenin and imipramine on the number of locomotor counts. Data are expressed in mean ± SEM (n = 8). Data were evaluated using a one-way analysis of variance (ANOVA) and Tukey’s test. * *p* < 0.05 when compared to the vehicle control group. LA: low dose of Apigenin (25 mg/kg); HA: high dose of Apigenin (50 mg/kg); Imipramine: standard antidepressant (15 mg/kg); Vehicle; 0.2% carboxy methyl cellulose (CMC).

When compared to the group that received a vehicle treatment, animals given a high dose of apigenin and imipramine showed a significantly (*p* ˂ 0.05) higher locomotor count (Figure 3). On the other hand, animals who received a low dose of apigenin showed no noticeable change in their locomotor count.

### 2.4. Plasma Nitrite and Corticosterone

**Figure 4:**
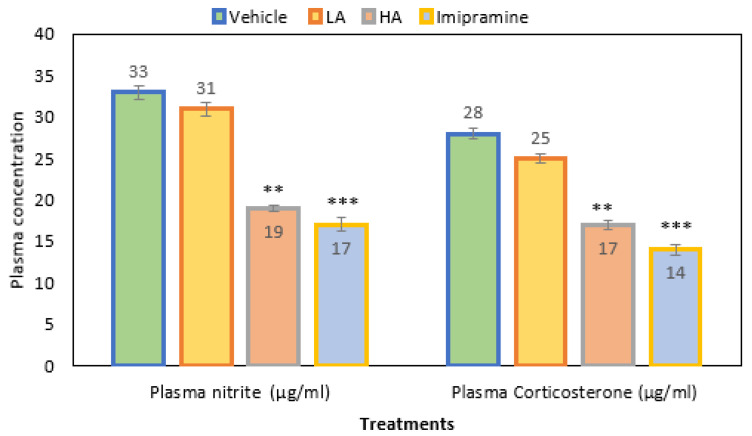
The effect of apigenin and imipramine changes on plasma nitrite and corticosterone levels. Data are expressed in mean ± SEM (n = 8). Data were evaluated using a one-way analysis of variance (ANOVA) and Tukey’s test. ** *p* < 0.01; *** *p* < 0.001 when compared to the vehicle control group. LA: low dose of Apigenin (25 mg/kg); HA: high dose of Apigenin (50 mg/kg); Imipramine: standard antidepressant (15 mg/kg); Vehicle; 0.2% carboxy methyl cellulose (CMC).

When apigenin was given at a high (*p* ˂ 0.001) dose, the plasma nitrite levels were much lower than in the group that received the vehicle treatment. As with a high dose of apigenin, the administration of imipramine significantly (*p* ˂ 0.001) decreased the plasma nitrite level (Figure 4). Overall, plasma nitrite levels decreased by 42% in a group that received a high dose of apigenin. In comparison, a 48% reduction was noticed with standard antidepressant imipramine and just 6% with low doses of apigenin when compared to the vehicle control group. 

Additionally, mice given a high dose of apigenin had a significantly (*p* ˂ 0.01) decreased plasma corticosterone level when compared to the vehicle control. Further, imipramine also produced a significant (*p* ˂ 0.001) decrease in plasma corticosterone, which was even more than the high dose of apigenin (Figure 4). Overall, the plasma corticosterone level decreased by 39% due to the administration of a high dose of apigenin, a reduction of 50% was observed in the imipramine group, and a decrease of only 10% was due to a low dose of apigenin when compared to the vehicle-treated control group. 

### 2.5. Brain Malondialdehyde (MDA) Levels

**Figure 5:**
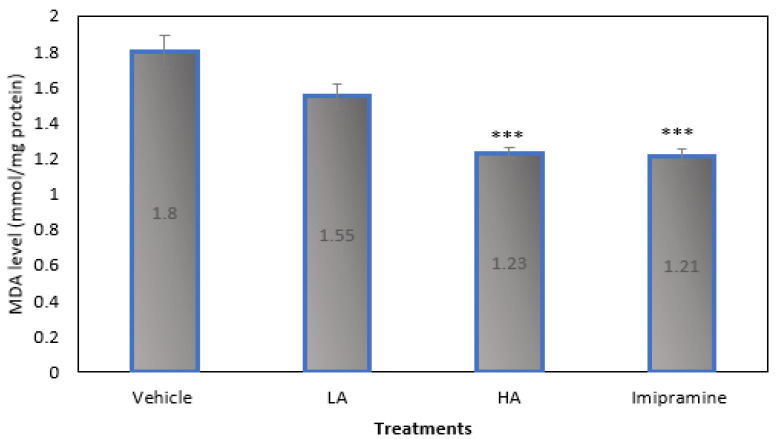
The effect of apigenin and imipramine on brain malondialdehyde (MDA) levels. Data are expressed in mean ± SEM (n = 8). Data were evaluated using a one-way analysis of variance (ANOVA) and Tukey’s test. *** *p* < 0.001 when compared to the vehicle control group. LA: low dose of Apigenin (25 mg/kg); HA: high dose of Apigenin (50 mg/kg); Imipramine: standard antidepressant (15 mg/kg); Vehicle; 0.2% carboxy methyl cellulose (CMC).

Figure 5 demonstrates that, when compared to the vehicle-treated group, mice given large doses of apigenin and imipramine after CMS had significantly (*p* ˂ 0.001) lower brain MDA levels. High doses of apigenin and imipramine reduced brain MDA levels in nearly identical ways.

### 2.6. Brain Catalase Activity

**Figure 6:**
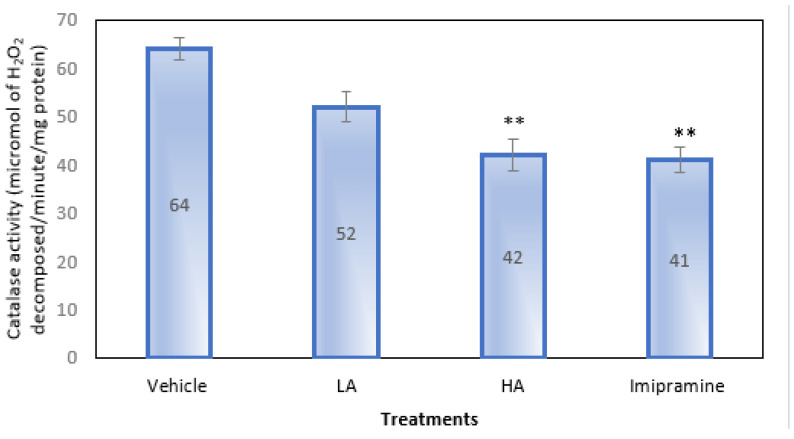
The effect of apigenin and imipramine on brain catalase activity. Data are expressed in mean ± SEM (n = 8). Data were evaluated using a one-way analysis of variance (ANOVA) and Tukey’s test. ** *p* < 0.01 when compared to the vehicle control group. LA: low dose of Apigenin (25 mg/kg); HA: high dose of Apigenin (50 mg/kg); Imipramine: standard antidepressant (15 mg/kg); Vehicle; 0.2% carboxy methyl cellulose (CMC).

Only a high dose of apigenin and imipramine, as shown in Figure 6, were able to significantly (*p* ˂ 0.01) lower the brain’s catalase activity as compared to the group that received a placebo. Even though apigenin at low doses reduced catalase activity, it was not significantly less.

### 2.7. Brain Glutathione Levels

**Figure 7:**
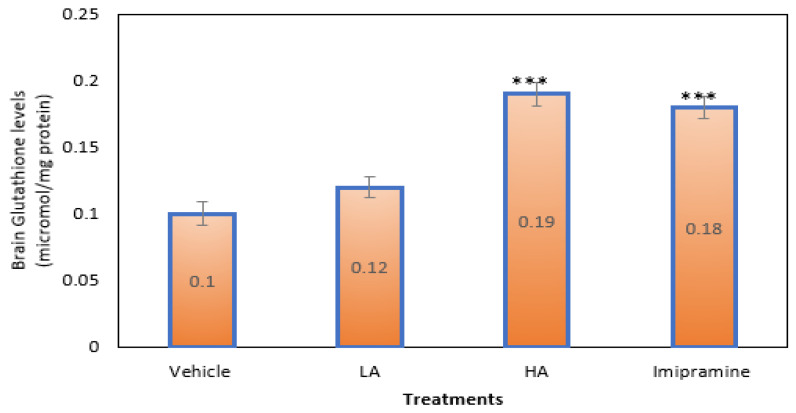
The effect of apigenin and imipramine on brain-reduced glutathione levels. Data are expressed in mean ± SEM (n = 8). Data were evaluated using a one-way analysis of variance (ANOVA) and Tukey’s test. *** *p* < 0.001 when compared to the vehicle control group. LA: low dose of Apigenin (25 mg/kg); HA: high dose of Apigenin (50 mg/kg); Imipramine: standard antidepressant (15 mg/kg); Vehicle; 0.2% carboxy methyl cellulose (CMC).

Those mice that were given a high dose of apigenin plus the conventional antidepressant imipramine had significantly (*p* ˂ 0.001) higher amounts of SGH in their brains than animals shown a vehicle. High doses of apigenin elevated brain SGH levels more than regular imipramine (Figure 7).

## 3. Discussion

The search for new antidepressants based on unique approaches may help develop more effective and efficient antidepressants. In searching for relatively safe and effective antidepressant drugs, recent research has given more weight to natural products [22,23]. This study aimed to examine the effectiveness of apigenin in treating depression brought on by chronic mild stress in an animal model system.

The outcome of this study shows that apigenin has the dose-dependent property to decrease depression, which is possibly due to its antioxidant potential. The antidepressant effects of apigenin were comparable to those of imipramine, a common tricyclic antidepressant. Chronic moderate stress (CMS) is a paradigm widely used to induce depressed behavior in mice that matches the pattern of depression that people experience when subjected to multiple stressors during a typical day [24]. The tail suspension test (TST) and the sucrose preference test are two behavioral models that are frequently used to assess potential antidepressants [25]. Chronic mild stress significantly increased immobile time in the TST setup, but a high dose of apigenin and imipramine significantly reduced it. Additionally, imipramine and a high dose of apigenin greatly enhanced the locomotor activity of CMS mice when compared to the controls, which confirmed their effects as CNS stimulants. Previous research [18] has shown that the alpha-adrenergic receptor, 5 HT3, D1, D2, and D3 receptors, may have a role in the antidepressant effects of apigenin in rats.

The sucrose preference test was another method used to assess the antidepressant effects of apigenin on CMS mice. This test is designed to identify anhedonia-like behavior, which is characterized by a subject’s loss of interest in or enjoyment of generally joyful or cheery activities. It is one of the more prevalent signs of depression [26]. Mice had a clear preference for sucrose before CMS started. However, the percentage of sucrose preference significantly decreased in the three weeks following CMS. Apigenin treatment during CMS resulted in a dose-dependent reinstatement of sucrose preference. When compared to the control, a high dose of apigenin led to a recovery similar to that brought on by imipramine. However, a low dose was not able to increase sucrose preference significantly. This result indicates that apigenin’s antidepressant-like action is dose-dependent. Similar antidepressant efficacy of apigenin was demonstrated in a previous work utilizing male BALB/c mice that had undergone restraint stress by promoting autophagy via the AMPK/mTOR pathway. These findings indicated that apigenin is a potential antidepressant agent that works through multiple mechanisms to alleviate stress-induced depression.

When the hypothalamic-pituitary-adrenal (HPA) axis is activated, plasma glucocorticoid levels will alter, which may lead to depression [27]. According to Sousa et al. [28], high cortisol levels can impact various mental functions, including the emergence of depressive symptoms. The long-term use of antidepressants is known to lower HPA activity and return the HPA axis to a healthy condition [28]. Other studies have found that modest chronic stress increases plasma corticosterone levels by hyperactivating the HPA [29]. The hyperactivity of the HPA axis caused by CMS in stressed mice was reduced by high doses of apigenin and imipramine treatments, as shown by a sharp decrease in plasma corticosterone levels in the stressed animals. Our results are consistent with a previous study that showed apigenin could reverse the depression-like behavior that corticosterone induced in mice [30].

The stress produced by CMS causes the body to generate oxygen free radicals, which are shown by increased blood nitrite levels [31]. Plasma nitrite levels were decreased in mice given a high dose of apigenin during CMS, which indicated a decrease in nitrosative stress. The oxidative damage that CMS inflicted on the mice was effectively prevented by apigenin, which is a characteristic of many natural substances [32].

Oxygen-derived free radicals play a role in depression. By activating immune-inflammatory processes and abnormalities in lipids, reactive oxygen species production, lipid peroxidation, and impaired antioxidant enzyme activities may result [33]. These processes may also be connected to depression. The antioxidant capacity of the brain is diminished by CMS, which is possibly due to the production of reactive oxygen species [34]. CMS decreased glutathione levels in the brain while increasing lipid peroxidation and catalase activity. Following three weeks of apigenin treatment, these traits were noticeably reversed. Considering this, apigenin revealed potent antioxidant activity in mice. In mice exposed to CMS, plasma nitrite levels were significantly increased [35]. Apigenin significantly decreased nitrosative stress in stressed mice by reducing plasma nitrite levels. Apigenin, therefore, significantly protected the mice from oxidative damage caused by CMS. As a result, we hypothesize that apigenin may act as an antidepressant in animal models at high doses through several different pathways. The HPA axis will most likely be restored, the antioxidant potential will be increased, and free radicals will be eliminated. Although apigenin was found to be effective at doses of 25 mg/kg in several earlier studies [16,35], we were only able to detect a substantial benefit at 50 mg/kg. This is likely because our investigation used a chronic stress paradigm, which requires a strong and robust antidepressant agent that can neutralize the stress-inducing chemicals and stabilize the body against toxic metabolites. The findings of this study, therefore, suggest that apigenin functions as an antidepressant by reducing stress and scavenging oxidative free radicals, in addition to its capacity to replenish depleted neurotransmitters and prevent inflammation. 

## 4. Materials and Methods

### 4.1. Study Samples

Albino mice (20–25 g, 10–14 weeks old) were maintained in an air-conditioned space using standard animal care procedures. All factors were kept constant during the experiment to avoid influencing the animals’ behavioral habits. They were fed standard animal pellets procured from a nearby supplier. The institutional ethics committee approved the study’s proposal. 

### 4.2. Chemicals and Reagents

Sigma-Aldrich provided the imipramine hydrochloride [≥99% (TLC)]. Apigenin was supplied by Beijing Mesochem Technology Co., Ltd. (Beijing, China) (purity: 98.20 percent by HPLC). The remaining substances needed for this research came from reputable vendors, and the labels were saved for later reference. The doses of apigenin [16] and imipramine [36] were selected based on our earlier findings. 

### 4.3. Experimental Grouping

**Figure 8:**
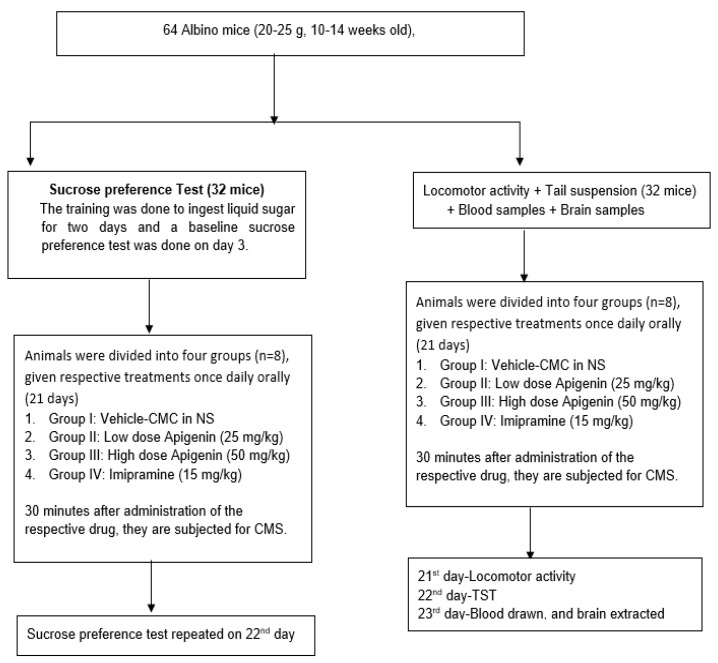
Experimental protocol of the study.

Eight groups, each including eight mice, were formed from all the mice used in the study (Figure 8). All treatments were done orally and once daily. As a vehicle, 0.2% carboxymethyl cellulose (CMC) in 0.9% sodium chloride was administered to the group I, while dosages of apigenin of 25 mg/kg (low dosage of apigenin—LA) and 50 mg/kg (high dose of apigenin—HA) were administered to groups II and III, respectively. Animals in group IV were given imipramine (15 mg/kg). For three weeks, all treatments (chronic mild stress—CMS) were ingested orally 30 min before the onset of stress (21 days). The mice’s locomotor activity was measured on day 21 following 60 min of stress exposure. The mice underwent a TST on the 22nd day. Vehicle therapy (CMC) was given to the animals in group V, whereas an LA and HA were provided to the animals in groups VI and VII, respectively. Group VIII animals received imipramine (15 mg/kg). All treatments were given orally for three weeks before subjecting the animals to their respective stress pattern of CMS. A sucrose preference test was conducted on day 21 at 60 min following CMS [36].

### 4.4. Chronic Mild Stress (CMS)

Neuronal toxicity brought on by ongoing stress may cause depression. The mice were exposed to mild chronic stress with a method that has been previously described [37,38]. For three weeks, mice were subjected to one daily stress pattern. The prescribed drugs were administered half an hour before subjecting the animals to specific stresses. On the first week of CMS, animals were immobilized for 2 h on day 1, exposed to empty water bottles for 1 h on day 2, kept in the dark at night on day 3, exposed to foreign bodies for 24 h on day 4, had their cages tilted to 45 degrees for 7 h on day 6, and had their tails pinched for 30 s on day 7. The order of the stress patterns changed continually.

### 4.5. Tail Suspension Test

The established tail suspension test (TST) was used as per the details fully described earlier [39]. 

### 4.6. Measurement of Locomotor Activity 

The ratings of control and the test animals’ horizontal locomotor activity were obtained using a method suggested by Dhingra et al. [39].

### 4.7. Sucrose Preference Test

Animals were trained to ingest liquid sugar while fasting for two days before being subjected to continuous mild stress. The mice were given two bottles three days later, following a 23 h fast, with one containing ordinary water and the other a sucrose solution. The baseline preference for sucrose solutions was calculated [37]. To determine the effect of the therapy on the animals’ preference for sucrose solution as a percentage—which served as a marker for depression brought on by stress—the test was repeated after 21 days of therapy.

### 4.8. Nitrite and Corticosterone Measurements 

On day 23, blood was drawn from the retro-orbital, and plasma was separated using a centrifuge to assess the levels of nitrite and corticosterone [40,41]. This was carried out an hour after the therapy [36].

### 4.9. Reduced Glutathione and Catalase Activity 

The mice were decapitated on the 23rd day, and their brains were extracted after blood samples were obtained. A cold buffer (pH 7.4) made up of 0.25 M sucrose, 0.1 M Tris, and 0.02 M ethylenediamine tetraacetic acid was used to wash the acquired brain samples. Samples of the brains were centrifuged. The catalase levels, reduced glutathione, and lipid peroxidation were assessed in the centrifuged supernatants. The Wills technique [42] and a UV-visible spectrophotometer were used to measure malondialdehyde levels and identify lipid peroxidation. The method of Jollow et al. [43] was utilized to measure reduced glutathione, and catalase activity was quantified by the Claiborne method [44] using a UV-visible spectrophotometer.

### 4.10. Analysis of Results

Eight animals from each group were used to collect the data needed for the analysis. The data were evaluated using a one-way analysis of variance (ANOVA) and Tukey’s test (GraphPad Prism). Data were presented as mean ± SEM in the tables, and differences were regarded as significant when the *p*-value for comparison between different groups was less than 0.05.

## 5. Conclusions

The findings of this study suggest that, at high doses, apigenin may have antidepressant properties that are similar to the commonly used antidepressant imipramine. Due to a rise in brain glutathione levels, apigenin may have antidepressant effects, in addition to its antioxidant and corticosterone restoration capabilities. However, more investigation is required to determine whether neurotransmitters and other pathways play a part in the antidepressant effects of apigenin.
